# Effect of a simple core muscle training program on trunk muscle strength and neuromuscular control among pediatric soccer players

**DOI:** 10.1186/s40634-021-00353-y

**Published:** 2021-05-06

**Authors:** Ryotaro Kumahara, Shizuka Sasaki, Eiji Sasaki, Yuka Kimura, Yuji Yamamoto, Eiichi Tsuda, Yasuyuki Ishibashi

**Affiliations:** 1 Department of Orthopaedic Surgery, Hirosaki University Graduate School of Medicine, 5 Zaifu-cho, Hirosaki, 0368562 Japan; 2 Department of Rehabilitation Medicine, Hirosaki University Graduate School of Medicine, Hirosaki, Japan

**Keywords:** Core muscle training, Pediatric athletes, Anterior cruciate ligament injury prevention

## Abstract

**Purpose:**

The purpose of this study was to examine the effect of simple core muscle training (CMT) program on trunk muscle strength and neuromuscular control among pediatric athletes.

**Methods:**

Forty-nine male soccer players (mean age, 10.8 years) participated. The CMT program had three components (bench, side bench, and Nordic hamstrings) and was performed at least three times weekly for a year. Trunk flexion/extension muscle strength and the K/H ratio (determined by dividing knee separation distance by hip separation distance during drop-jump test and used as an index of lower limb valgus alignment) were measured, and the Y balance test (YBT) was performed before and after intervention. This study did not include the pure control group among the same team. To consider the effect of CMT on trunk muscle strength due to physical growth, we used the data of trunk muscle strength from the local cohort study previously conducted in our institution. One hundred participants who matched the age, height, body weight, and body mass index of the training group was designated as a control group.

**Results:**

In the training group, the trunk flexion/extension strength significantly increased at 6 months (*p* < 0.001, *p* < 0.001, respectively) and 12 months (*p* < 0.001, *p* < 0.001, respectively) compared to initial value. The K/H ratio at initial contact and maximum knee flexion phase significantly increased at 6 months (*p* < 0.001 and *p* < 0.001, respectively); however, it did not increase at 12 months (*p* = 0.384 and *p* = 0.070, respectively) compared to the initial value. In the YBT, the maximized reach distance in each direction significantly increased after intervention on both the dominant and non-dominant sides, except in the posteromedial direction on the non-dominant foot. Compared to the control group, although there was no significant difference in trunk flexion/extension strength at baseline (*p* = 0.141 and *p* = 0.390, respectively), the training group showed significantly higher trunk flexion/extension muscle strength at 12 months (*p* < 0.001 and *p* < 0.001, respectively).

**Conclusion:**

The CMT program increased trunk muscle strength and improved dynamic balance among pediatric male athletes.

**Level of evidence:**

Level II

## Introduction

The incidence of anterior cruciate ligament (ACL) injury in the pediatric population has increased by 2.3% over the last 20 years [2]. It was reported that very few injuries were found in patients < 10 years old; however, the incidence of ACL injury extremely increased at age 10–14 years [12]. Skeletally immature athletes demonstrate risky movement patterns such as dynamic knee valgus and decreased knee flexion during vertical and lateral jump landings [15, 33]. In addition, ACL reconstruction for skeletally immature patients has a risk of postoperative growth disturbance or residual deformity [3, 5]. Therefore, prevention of ACL injury in this age group is considered crucial.

A recent systematic review concluded that ACL injury prevention programs have a significant protective effect [18]. However, it is difficult to adapt complex and multifactorial training for young athletes [8]. For successful interventions in this age group, an efficient program that demonstrates high compliance in this age group should be developed [20].

Most ACL injuries occur by noncontact mechanism, including landing from a jump, cutting, and decelerating during sporting activities [14, 23], and it has been reported that increasing knee valgus angle and moment are predictive factors [16]. Hewett et al. [17] reported that these four neuromuscular imbalances contribute to noncontact ACL injury: quadriceps dominance (stabilizer of knee joint), leg dominance (side-to-side asymmetry of the lower extremities), ligament dominance (anatomic and static stabilizers to absorb the ground reaction force), and trunk dominance (inability to precisely control the trunk in three-dimensional space). These factors can theoretically be modified by appropriate interventions. Core stability exercise is one of the most favored components that are frequently included in ACL injury prevention programs. It was reported that core stability predicted the risk of ACL injury [35]. Moreover, Sasaki et al. [29] reported that an 8-week core muscle training (CMT) reduced knee valgus moment during jump landing task among collegiate female basketball players. Their CMT program consisted of three components and could be performed in a short time; however, its effect on the pediatric population remains unclear.

This study primarily aimed to evaluate the effects of a simple CMT program on trunk muscle strength and neuromuscular control among pediatric soccer players over time. The secondary purpose was to compare the trunk muscle strength with matched control who participated in sports activity but did not perform simple CMT program, considering the effects of physical growth. We hypothesized that a simple CMT program would strengthen the trunk muscles and lead to improvements in lower limb biomechanics and dynamic balance.

## Materials and method

### Participants

Sixty-seven male soccer players aged 9–13 years who belonged to a local soccer club participated in this prospective study. The following were excluded: participants who had a surgical history involving the lower limbs; those who could not be followed up after 6 months and 1 year; and those who rested for more than 1 month due to injuries during the intervention period. The study design was approved by the ethics committee of our institution, and informed consent was obtained from the coaching staff and all subjects’ parents.

### Intervention

**Fig. 1:**
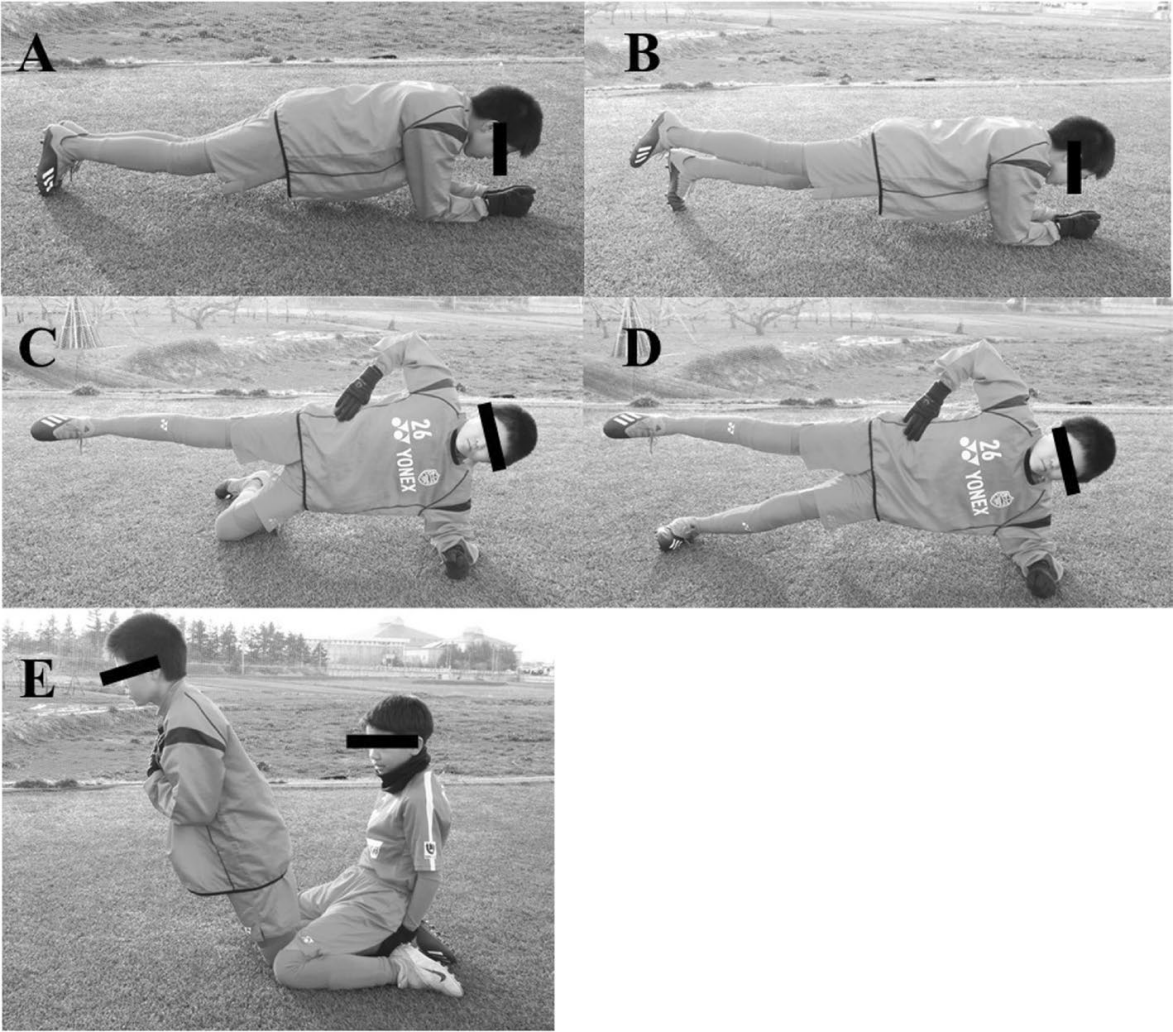
Core muscle training program (**a**-**e**)

Based on a previous report, the CMT program consisted of only three components: bench (plank), side bench (side plank), and Nordic hamstrings [29] (Fig. 1, Table 1). We instructed the coaching staff on the training method. The CMT session was performed in about 5 min before the regular practice, at least 3 times per week for a year (both during the season and pre-season). CMT intensity was set to 2 or 3 levels and increased according to the degree of each participant’s achievement (Table 1). To ensure training compliance of the subjects, the training was monitored once every 2 weeks by the first author. Furthermore, we kept in close contact with the coaching staff and confirmed that the participants were training at least three times a week.

**Table 1:** Core muscle training program

Traning program	Repetitions	
Bench		
Level 1: static (both legs)	2 × 30 s	Fig. 1a
Level 2: 1 leg lift	2 × 30 s	Fig. 1b
Side Bench		
Level 1: static	2 × 30 s	Fig. 1c
Level 2: with leg lift	2 × 30 s	Fig. 1d
Hamstrings		Fig. 1e
Level 1: low load	3–5 times	
Level 2: middle load	5–10 times	

### Outcome measure

Trunk muscle strength was measured with a portable trunk muscle torque measurement instrument (PTMI) [27]. The PTMI is loaded with a load cell and consists of a simply fixable seat and torque stand. The subjects were fixed by three adjustable belts to prevent rotation of the pelvis and minimize the influence of the iliopsoas, quadriceps, and gluteus maximus. The seat could be rotated 180 degrees, in which measurements of both flexion and extension torque could be measured. The peak torque of isometric contraction strength of trunk flexion and extension was measured and normalized to body weight (in kg).

Dynamic control of the lower limb was evaluated with the drop-jump screening test (DJT) in accordance with the procedure previously described by Noyes et al. [22]. The participants took their shoes off and were instructed to drop off a 35-cm-high box, land on both feet on the floor, and then immediately perform a maximum vertical jump. Each participant practiced several times. No instructions were given on the position of the lower limbs and trunk, to avoid coaching effect on the subjects’ performances. The DJT was performed three times. The image data were simultaneously recorded with a digital video camera (HDR-HC3, Sony, Japan) at a sampling rate of 30 Hz. The camera was placed on a 100-cm-high camera stand, 4 m from the front face of the box. For the two-dimensional (2D) motion analysis, reflective markers with diameter of 25 mm were secured at the greater trochanter (hip marker) and the center of the patella (knee marker) on both legs. Two images were captured as still photographs at the following time points: initial contact (IC), defined by the frame in which the subject’s toes just touched the ground after dropping off the box, and maximum knee flexion (MKF), defined by the frame in which the hip markers reached the deepest point after IC. The separation distance between the two hip markers and that between the two knee markers were measured on the still images of the IC and MKF. The separation distance was measured in 1/100 mm units on the computer display, by appropriate setting of the computer software (Image J, National Institutes of Health). The knee separation distance was divided by the hip separation distance to assess the control of the lower limbs in the coronal plane and was defined as the K/H ratio in this study [28]. This ratio is an index of lower limb valgus alignment. The average of the three measurements was calculated.

The Y balance test (YBT) is a reliable tool for predicting injury risk, and it was reported that the intratester and intertester reliability were 0.85–0.89 and 0.97–1.00, respectively [25]. YBT was conducted in the same way as in previous reports [4, 6, 13, 25, 26]. Initial testing included the measurement of leg length, which was measured from the most distal aspect of the anterior superior iliac spine to the most distal aspect of the medial malleolus for each limb. The YBT reach directions were evaluated by affixing three tape measures to the floor, which we oriented anterior to the apex, and two aligned at 135° to this in the posteromedial and posterolateral directions. All tests were conducted with the study subjects barefoot. Participants received demonstrations and verbal instructions and were tested after the practice sessions. They were instructed to perform maximal reaches with the non-stance limb in the three directions while maintaining a single-leg stance on the test limb and conducting three formal test trials in each direction for both legs. The trial was judged invalid if the participant removed his hands from his hips, did not return to the starting position, applied sufficient weight through the reach foot to gain an increase in reach distance, raised or moved the stance foot during the test, or kicked the floor with the reach foot to gain more distance. Reach distances were normalized to limb length by calculating the maximized reach distance (%MAXD) using the following formula: excursion distance/limb length × 100 [6]. The dominant leg was determined by asking which leg they could kick a ball farther.

The incidence of sports injuries during the intervention period was investigated with a self-administered questionnaire.

### Statistical analysis

All data were measured at three time points: initial evaluation, after 6 months, and after 12 months. The normality was tested using the Shapiro–Wilk test, and the primary outcome, trunk flexion, and extension strength at baseline in the training group was normally distributed (p = 0.780 and 0.283, respectively). Moreover, repeated-ANOVA test with the α level set at 0.05 was used for comparing the overall data of each measurement before and after CMT training. Additionally, paired t test as a post hoc analysis was performed on the data of repeated-ANOVA test, with a significant difference between initial evaluation and at 6 months, initial evaluation and at 12 months. We followed the adjustment of Bonferroni correction and defined p < 0.017 as a significant difference.

In order to achieve 80% statistical power with an alpha of 0.05, power analysis revealed that a minimum of 12 participants would be required for detecting differences in mean value of trunk muscle strength in total samples with paired t test. In this analysis, the calculated effect size was 0.901, with 0.71 standard deviation.

This study did not include an untrained control group within the club. In order to consider the changes in trunk muscle strength due to physical growth, we used the data of trunk muscle strength from the Iwaki cohort study conducted in our institution. The Iwaki study was a medical examination for the local general population, in which athletic ability or physical function was examined once a year [28]. A total of 514 male students aged 10–13 years participated in the study from 2013 to 2016. DJT and YBT were not evaluated during this period. Of the 514 participants, 266 (52%) belonged to the sports club and practiced at least three times a week. Among these 266, we extracted the data of 100 participants who matched the age, height, body weight, and body mass index (BMI) of the subjects of the present study and designated them as a control group (Table 2). The differences in trunk muscle strength between the training and control groups were examined with two-sample t test, and *p* < 0.05 was considered statistically significant.

**Table 2:** Demographic data of the training group and control group

	Training group, *n* = 49	Control group, *n* = 100	*P* value
Initial evaluation			
Age, y	10.8 ± 1.1	10.7 ± 0.5	0.700
Height, cm	144.0 ± 7.8	142.3 ± 6.7	0.173
Weight, kg	33.9 ± 5.7	34.0 ± 5.0	0.952
BMI, kg/m^2^	16.3 ± 1.7	16.7 ± 1.7	0.140
After 12 months			
Age, y	11.8 ± 1.1	11.7 ± 0.5	0.701
Height, cm	151.0 ± 8.1	150.2 ± 8.2	0.574
Weight, kg	39.8 ± 7.1	39.4 ± 6.2	0.729
BMI, kg/m^2^	17.3 ± 1.8	17.4 ± 1.8	0.836

Data are represented as mean ± standard deviation

*BMI* body mass index

All statistical analyses were performed with Statistical Package for the Social Sciences (version 27.0, SPSS, Inc.).

## Results

**Fig. 2:**
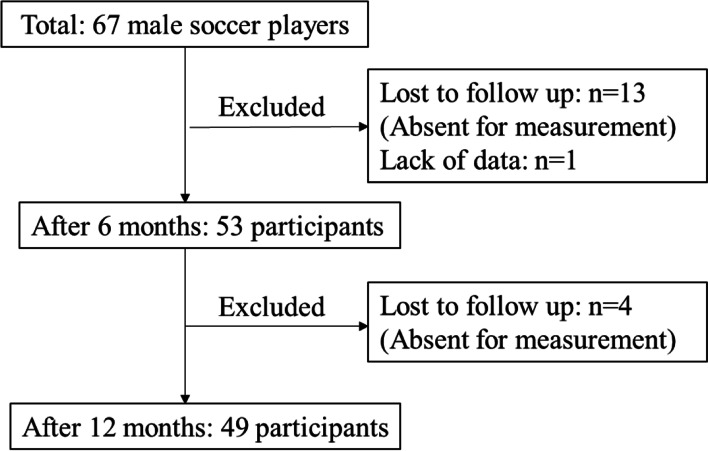
Flowchart of the training group

Forty-nine of sixty-seven subjects (73%) (mean age, 10.8 years) were followed up for 6 and 12 months (Fig. 2, Table 2). We confirmed that all participants could continue the CMT program at least three times a week during intervention period by detailed supervising and hearing from the coaching staff.

**Fig. 3:**
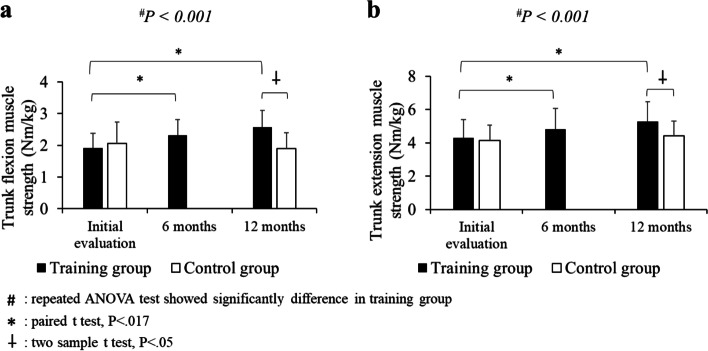
Comparison of normalized trunk flexion (**a**) and extension (**b**) muscle strength in the training and control groups. ^#^*P* < 0.01 in repeated ANOVA test, *P < 0.017 in paired t test, *P* < 0.05 in two-sample t test

In the training group, the trunk flexion strength significantly increased from 1.9 ± 0.5 Nm/kg to 2.3 ± 0.5 Nm/kg in 6 months (*p* < 0.001) and to 2.6 ± 0.5 Nm/kg in 12 months (*p* < 0.001). The trunk extension strength significantly increased from 4.3 ± 1.1 Nm/kg to 4.8 ± 1.2 Nm/kg in 6 months (*p* < 0.001) and to 5.3 ± 1.2 Nm/kg in 12 months (*p* < 0.001) (Fig. 3).

The K/H ratio at IC significantly increased at 6 months (*p* < 0.001), but not at 12 months (*p* = 0.384) compared to the initial value. The K/H ratio at MKF significantly increased at 6 months (*p* < 0.001), but not at 12 months (*p* = 0.070) compared to the initial value (Table 3).

**Table 3:** Total data of initial evaluation and after 6 and 12 months in the training group

	Initial evaluation	After 6 M	After 12 M
K/H ratio			
IC, %	0.89 ± 0.17	1.02 ± 0.16*	0.87 ± 0.14
MKF, %	0.66 ± 0.25	0.82 ± 0.23*	0.72 ± 0.17
%MAXD			
Dominant foot			
Anterior, %	95.2 ± 11.7	120.1 ± 18.6*	110.1 ± 14.5*
Posterolateral, %	86.3 ± 13.5	116.9 ± 14.7*	117.5 ± 14.2*
Posteromedial, %	97.9 ± 13.3	114.0 ± 27.2*	108.9 ± 20.2*
Non-Dominant foot			
Anterior, %	96.7 ± 13.7	123.0 ± 21.0*	113.0 ± 15.2*
Posterior Lateral, %	86.2 ± 13.3	116.4 ± 16.8*	118.2 ± 16.6*
Posterior Medial, %^a^	100.9 ± 18.1	114.3 ± 28.9	109.4 ± 21.7

Data are represented as mean ± standard deviation

*K/H ratio* Knee separation distance divided by hip separation distance, *IC* Initial contact, *MKF* Maximum knee flexion, *%MAXD* Maximized reach distance

^*^P < 0.017, significant difference after comparing with value at initial evaluation

^a^Data of Posterior Medial was not significantly different by repeated- ANOVA test, so paired t test was not performed

In the YBT, %MAXD in each direction significantly increased after 6 months and 12 months on both the dominant and non-dominant sides compared to the initial value, except for %MAXD in the posteromedial direction on the non-dominant foot (Table 3).

In the control group, the values of trunk flexion and extension strength were 2.1 ± 0.7 Nm/kg and 4.1 ± 0.9 Nm/kg, respectively, at the initial evaluation, and there was no significant difference when compared with the training group (*p* = 0.141 and *p* = 0.390, respectively). The values of trunk flexion and extension strength in the control group 12 months later were 1.9 ± 0.5 Nm/kg and 4.4 ± 0.9 Nm/kg, respectively, and they were significantly lower than the values obtained in the training group (*p* < 0.001 and *p* < 0.001, respectively) (Fig. 3).

Within the intervention period, there was no occurrence of injuries that would interrupt CMT training for more than 1 month, and no ACL injuries were observed.

## Discussion

The most significant finding of this study was that the CMT program in 12 months had significant effects on strengthening the trunk muscles, even for pediatric athletes. Moreover, this improvement of core stability seemed to lead to the modification of lower limb biomechanics and dynamic balance.

Sasaki et al. [29] reported that a simple CMT program may reduce the risk of noncontact ACL injury among collegiate athletes. After the 8-week simple CMT program, neuromuscular control and muscle strength of the lower limb and trunk were modified in their study. Junker et al. [19] reported that 8 weeks of core-stability training improved dorsal trunk strength for athletes aged 18–48 years. In addition, hamstring flexibility was improved by performing core-stability training. To our knowledge, few reports have verified the effectiveness of CMT program alone on trunk muscle strength, lower limb biomechanics, and dynamic balance in pediatric athletes. The subjects of this study were going through a growth spurt. Even considering the change in muscle strength due to growth, this study demonstrated significant strengthening of the trunk muscles in the training group compared to the control group. Therefore, our results indicated that the simple CMT program during intervention period had muscle-strengthening effect.

The K/H ratio is an index of dynamic lower limb alignment and has been shown to correlate with the knee valgus angle in 3D motion analysis [28]. In this study, the K/H ratio after CMT program significantly increased after 6 months, but not after 12 months, during IC and MKF. The results of K/H ratio after 6 months during IC and MKF indicate improvement in dynamic valgus alignment. Several previous reports have revealed a relationship between trunk and lower limb biomechanics.

The K/H ratio at IC and MKF did not change significantly between the initial value and after 12 months because the subjects of this study were only males. It has been reported that female athletes showed larger valgus angle and moment during jump landing task [16, 28]. Therefore, there was little potential for improvement of valgus alignment in male athletes, and it is possible that no significant change was observed. In addition, it was difficult to accurately evaluate knee kinetics for landing because 3D motion analysis was not performed in this study. Koga et al. [21] reported that sudden knee valgus moment and noncontact ACL injury occurred approximately 40 ms after initial contact in 3D motion analysis. Sasaki et al. [29] reported that CMT program improved the peak of the knee valgus moment after initial contact. Since this study used the same program as them, it was possible that there were kinetic changes that could not be detected by 2D motion analysis.

Our results showed significant increase in %MAXD in all directions after 6 and 12 months in YBT. YBT has been reported to be a reliable measure of postural control in younger athletes [13]. Balance training has been incorporated in several previous sports injury or ACL injury prevention programs [11, 24, 30]. Domingues et al. [9] reported that knee flexor strength correlated with dynamic balance ability, using a modified star excursion balance test. In addition, it was reported that core exercise improved lower limb strength balance [7]. The improvement in dynamic balance after the CMT program in this study is consistent with the results of these previous reports.

There is no consensus on when ACL injury prevention intervention should be applied. However, it is considered to be suitable for the pediatric population because the incidence of ACL injury has been shown to grossly increase from 10 to 14 years of age [12]. In recent years, various efforts have been made to prevent sports injury, including ACL injury, among pediatric athletes [1, 8, 10, 20, 31, 32]. Some studies concluded that prevention program had protective effects against sports injury [1, 10, 31]. In contrast, it was reported that there was no injury prevention effect [32]. One of the challenges of interventions for pediatric athletes is low compliance [10, 32]. Soligard et al. [31] suggested that high compliance was significantly related to decreased injury risk among 13–17-year-old athletes. Distefano et al. [8] reported that traditional injury prevention programs did not result in changes in biomechanics, whereas a prevention program designed for pediatric population modified knee kinematics.

It is necessary to develop programs that demonstrate higher compliance in this special age group. The CMT program used in this study can be performed in about 5 min; it was possible to incorporate it into daily team practice three times in a week and to continue training for 12 months. From the results of this study, simple CMT program alone had the effect of improving neuromuscular control.

Our study had some limitations. First, our control group was not constituted within the same team as the training group. In order to evaluate the training effect based on growth, the control group was established from the cohort study conducted in our institution. While the control group was active in sports, there were likely differences in how each sport trains and plays that could have affected a comparison of changes in trunk muscle strength and neuromuscular control. Therefore, it is difficult to conclude that the results of this study were truly the effects of the simple CMT program for the pediatric athletes. However, strengthening of trunk muscles was obvious when our subjects were compared with the matched control group that participated in sports, and it seemed that simple CMT program may improve core stability. Second, the subjects of this study were only male athletes and soccer players. The risk of ACL injury has been found to be higher in female soccer players than in their male counterparts [34]. Whether this simple CMT program will exert the same training effect in female athletes or other sports players is unknown. However, the sex difference in the incidence of ACL injury becomes evident after puberty [12]. Therefore, preventive intervention for male athletes is necessary in this age group. Moreover, there were no ACL injuries in the intervention period; however, this study institution was too short to assess whether there was an actual preventive effect of ACL injuries. A long-term intervention is considered to be necessary to clarify the effect of the CMT. Third, kinematic evaluation was performed by 2D motion analysis; hence, it was not possible to measure the knee moment. It is difficult to evaluate the biomechanics of landing using only 2D motion analysis; however, 3D motion analysis could not be performed because this study targeted a relatively large population, and 3D motion analysis required large equipment.

## Conclusion

Our results suggested that simple CMT program for 12 months had the effects of increasing trunk muscle strength and improving dynamic balance. The dynamic lower limb alignment during DJT improved after 6 months; however, there was no significant change at 12 months compared with the initial value. In addition, compared with the matched control group, the trunk flexion/extension muscle strength were significantly higher in the training group after intervention.
